# Microbial Diversity and Composition Uncovered on Obturator Prosthesis Biofilms: Exploratory Findings from a Pilot Study

**DOI:** 10.3390/pathogens15020221

**Published:** 2026-02-16

**Authors:** Camila Vilela, Leonel Mendoza, Raquel Vilela, Francisca Daniele Moreira Jardilino, Cláudia Lopes Brilhante Bhering, Amalia Moreno

**Affiliations:** 1 Department of Dental Clinic, Pathology and Surgery, Faculty of Dentistry, Federal University of Minas Gerais, Belo Horizonte 31270901, MG, Brazilfranciscadaniele.jardilino@gmail.com (F.D.M.J.);; 2 Department of Clinical and Toxicological Analysis, Faculty of Pharmacy, Federal University of Minas Gerais, Belo Horizonte 3127090, MG, Brazil; 3 Biomedical Laboratory Diagnostics, Microbiology, Genetics, and Immunology, Michigan State University, East Lansing, MI 48824, USA; 4 Department of Restorative Dentistry, Faculty of Dentistry, Federal University of Minas Gerais, Belo Horizonte 31270901, MG, Brazil

**Keywords:** metagenomic analysis, obturator prostheses, biofilms, microbial diversity, oral microbiome

## Abstract

Microbial communities on obturator prosthesis biofilms have yet to be investigated. This pilot study explores eukaryotes, prokaryotes, and viruses present on obturator prosthesis biofilms using metagenomics. The prostheses of the selected patients (*n* = 3) were collected and their biofilms were physically removed. The total genomic DNA was extracted, followed by metagenomic analysis. The microbial diversity in each of the investigated biofilms was exceptionally abundant. Between 2616 to 3024 species were detected in the three biofilms. The highest percentage included prokaryotes and unclassified species, followed by low percentages of fungi, viruses, and archaea. Unusual pathogens rarely reported in oral biofilms, such as *Mycobacterium* and other species, were also found at very low percentages. Unigenes for functional pathways related to metabolism, cellular processes, human disease, and other microbial unigenes were abundant. In addition, unigenes for several antibiotic-resistance mechanisms were also detected. This study reveals, for the first time, that biofilm formation on obturator prostheses comprises a variety of dynamic microbial communities, suggesting a putative role in health and disease in patients following maxillofacial surgery.

## 1. Introduction

The oral microbiome is one of the most complex communities, comprising a collection of eukaryotes, prokaryotes, and viruses [1,2,3,4]. The classic study by Marcotte and Lavoie 1998 [5] found that resident microbiota can undergo changes imposed by environmental pressure. Therefore, each host undergoes variations in the composition of their microbiota according to the oral environment present at a specific time. Zhang, 2018 [3] and Wade, 2012 [4] reported that, due to its diversity, the human oral microbiota is believed to be the second most complex anatomical site. Currently, the oral cavity is known to have a large microbiota composed of approximately 800 microbial species, with bacteria representing the largest percentage, followed by fungi, archaea, and bacteriophages, of which only 350 of these species can be recovered in culture [1,3,6,7]. These microbial communities live in homeostasis with each other, until the symbiotic system comes under pressure by extrinsic or intrinsic factors [3,6,7]. Individuals with compromised immune systems and/or undergoing invasive therapy could display changes in the composition of their normal microbiota that may contribute to the worsening of their original clinical conditions [2,8,9]. These types of resections were classified by Aramany [9], and later refined by Brown and Shaw [8], based on the extent of structural loss in both horizontal and vertical dimensions.

In the context of maxillofacial rehabilitation, obturator prostheses represent a unique ecological niche for microbial communities. Unlike transient matrices, such as saliva or oral swabs, dentures and obturators are long-term intraoral devices repeatedly exposed to saliva, dietary substrates, mucosal surfaces, and—after maxillectomy—to altered anatomy that may create communication between adjacent cavities [10]. Prolonged wearing times, retentive surfaces, and hygiene challenges can favor biofilm maturation and the persistence of low-abundance taxa [8,9,11,12,13,14,15]. Therefore, characterizing obturator-associated biofilms is clinically relevant to understanding potential reservoirs of opportunistic pathogens and antimicrobial resistance determinants in a vulnerable population. Little is known about biofilm formation on obturator protheses; thus, the main objective of this study was to identify eukaryotic, prokaryotic, and viral communities residing on the obturator prostheses biofilms using a deep metagenomic approach.

## 2. Materials and Methods

### 2.1. Study Design

This was a preliminary observational, cross-sectional study designed to characterize the taxonomic composition and functional potential of biofilms formed on maxillary obturator prostheses using shotgun metagenomics. Clinical variables included sex, age, defect classification [8], and dentition status. Metagenomic variables included taxonomic abundance profiles, alpha diversity metrics, the number of unigenes/species-level assignments, functional annotation (e.g., KEGG- https://www.genome.jp/kegg - /eggnog - http://eggnog5.embl.de/#/app/home - /CAZy - https://www.cazy.org/), and the detection of antimicrobial resistance genes (ARGs - https://www.cd-genomics.com/microbioseq/resource-args-detection-database-bioinformatics-tools.html).

### 2.2. Participants Prostheses

The participants were recruited from a total of 15 patients from the Oral and Maxillofacial Prosthesis Extension Clinic of the UFMG, School of Dentistry. Only 20% (*n* = 3) of the participants agreed to collaborate with the study. The remaining 80% argued that their privacy would feel violated due to the removal and sharing of a private part of their bodies and the physical discomfort that such action entails. Additional inclusion criteria required patients who did not use antimicrobials or other factors that could impact the survival or multiplication of microorganisms in their oral cavities. At the time of sampling, all participants had been wearing their obturator prostheses for at least 1 year (Table 1). All prostheses were removable partial obturators incorporating acrylic resin and a metal framework; all participants were partially dentate. The three selected patients followed regular hygienic oral care habits such as broaching their teeth and prosthesis after meals. Regarding diet habits, in general, Brazilian have a rich diet, including vegetables, several types of meat, rice, and beans. The three selected patients confirmed that the above meals were routine in their homes. At the time of collection, the presence of biofilms was physically observed on all prostheses (Table 1). According to the patients’ recollections, the prostheses had been used without being cleaned for more than 72 h before DNA extraction and metagenomic analysis.

### 2.3. Sample Collection

A standard methodology to collect biofilms on the surfaces of obturator prosthesis for DNA analysis is not available. Therefore, we developed a methodology to accurately collect the biofilms present on each of the investigated prostheses for DNA analysis. The collection started with the removal of prostheses from each of the investigated hosts, followed by placing the devices in 40.0 mL of sterile saline solution (SS). The biofilms present on the surfaces of the prostheses were physically removed using a universal curette; their contents were placed in the original 40.0 mL SS and then transferred to sterile 50.0 mL tubes, sealed, and transported on the same day to a molecular laboratory. After the biofilm’s removal, the prostheses were returned to their owners. At the laboratory, the 50 mL tubes were then centrifuged at 3000 g for 30 min. After centrifugation, the supernatant was removed and 100 µL of sterile distilled water was added to the pellet and thoroughly mixed.

### 2.4. Genomic DNA Extraction

The Fungi/Yeast genomic DNA extraction kit (NORGEN Biotek Corp., Thorold, ON, Canada) was used to process the microbial material present in each of the pellets containing the collected biofilms. The steps followed for genomic DNA extraction were as per the protocol provided by the manufacturer. The total DNA was eluted in 50 µL of elution buffer. After genomic DNA extraction, the DNA concentration in each sample was determined using a General Electric NanoVue UV/Visible instrument (General Electric Company, Piscataway, NJ, USA). The final DNA concentrations were as follows: RRRad = 5.1 µg/µL, CRRad = 4.85 µg/µL, and SFNRad = 4.75 µg/µL, respectively. Samples were then stored at −80 °C until further analysis.

### 2.5. DNA Library Construction, Whole Genomic Sequencing, and Metagenomic Analysis

Genomic libraries were constructed using the TruSeq™ DNA Nano Illumina library preparation kit (Illumina, Inc., San Diego, CA, USA). The final libraries were quantified with a Qubit 4.0 Fluorometer (Thermo Fisher Scientific Inc. Wilmington, NC. USA) using the DNA HS assay kit. Library insert size was assessed using a TapeStation system (Agilent Pvt. Ltd., New Delhi, India). Library concentration was determined by comparing the sample peak area with the known concentration of the upper marker. For sequencing, libraries were pooled and desaturated according to the standard workflow. The ExAmp/library mixture was then loaded onto the Illumina NovaSeq 6000 V1.5 flow cell (Illumina, Inc., San Diego, CA, USA); sequencing performance was monitored via BaseSpace “Sequence Hub” (https://www.illumina.com/products/by-type/informatics-products/basespace-sequence-hub.html, accessed on 16 May 2025). To evaluate quality, several primary metrics were considered, including the percent passing filter, aligned percentage, Q30/Q-score, and percent base (Illumina, Inc., San Diego, CA, USA).

### 2.6. Data Analysis

The libraries were prepared by throughput sequencing carried out using NovaSeq 6000, with the sequencing mode of PE150 (Illumina, San Diego, CA, USA). Raw sequencing reads were processed to obtain valid reads for further analysis. First, sequencing adapters were removed from sequencing reads using cutadapt v1.9 (https://cutadapt.readthedocs.io/en/v1.9.1/ accessed on 23 May 2025). Second, low-quality reads were trimmed by fqtrim v0.94 using a sliding-window algorithm (https://zenodo.org/records/20552, accessed on 23 May 2025). Third, to remove host DNA contamination, reads were aligned to the host genome using bowtie2 v2.2.0, which selects for human DNA reads to be deleted from the final microbial DNA (https://bowtie-bio.sourceforge.net/bowtie2/index.shtml, accessed on 23 May 2025). Once quality-filtered reads were obtained, they were de novo assembled to construct the metagenome for each sample by IDBA-UD v1.1.1 9 (https://github.com/loneknightpy/idba, accessed on 23 May 2025). Coding regions (CDS) of metagenomic contigs were predicted by MetaGeneMark v3.26 (https://genemark.bme.gatech.edu/license_download.cgi, accessed on 26 May 2025).

CDS sequences of all samples were clustered by CD-HIT v4.6.1 to obtain unigenes (https://github.com/weizhongli/cdhit/releases/tag/V4.6.1, accessed on 26 May 2025). Unigene abundance on the samples was estimated by TPM based on the number of aligned reads by bowtie2 v2.2.0 (https://sourceforge.net/projects/bowtie-bio/files/bowtie2/2.2.0/ accessed on 26 May 2025). The metaprodiga software was used to predict CDS (Coding Region) for the contigs (≥500 bp) assembled from each sample and filter out sequences with a CDS length of less than 100 nt, according to the prediction results (https://github.com/hyattpd/Prodigal/blob/GoogleImport/metagenomic.c, accessed on 28 May 2025). Subsequently, based on the CDS prediction results, we used the MMseqs2 software for redundancy removal to obtain a non-redundant unigenes set (https://github.com/soedinglab/MMseqs2, accessed on 26 May 2025). Lastly, we use Bowtie2/2.5.4 to align the CleanData sample to the unigene sequences, calculate the number of reads mapped to each unigene in each sample, and filtered out unigenes with the number of mapped reads ≤2 in all samples, thus obtaining the final unigenes for subsequent analysis (https://sourceforge.net/projects/bowtie-bio/files/bowtie2/2.5.4/ accessed on 27 May 2025). The calculation formula below (r = the number of reads mapped to the unigene, and L is the length of the unigene) was used to find the number of mapped reads and the length of each unigene, and to calculate the abundance information of the unigene in each of the samples. Species were defined as taxonomic units assigned via unigenes:

$$G_{k\;}\;=\;\frac{r_{k}}{L_{k}\;}\;\times \frac{1}{\sum_{i=1}^{n}\;\frac{r_{i}}{L_{i}}}\;\times \;10^{2}$$

### 2.7. Taxonomic and Functional Annotations

The obtained coding unigenes were translated into amino acid sequences and the lowest common ancestor taxonomy of the unigenes were obtained by aligning them against the National Center for Biotechnology Information (NCBI) NR database by DIAMOND v 0.9.14 (https://anaconda.org/bioconda/diamond) [16]. Similarly, the functional annotations (GO, KEGG, eggNOG, CAZy, CARD, PHI, MGEs, VFDB) of the unigenes were obtained.

### 2.8. Statistical Analysis

Based on the taxonomic and functional annotation of the unigenes, along with the abundance profile of the unigenes, differential analyses were carried out at each taxonomic or functional level by Fisher’s exact test (non-replicated groups) or the Kruskal–Wallis test (replicated groups). The chi-square test was applied to compare the number of eggNOG entries with decreased or increased abundances between the biofilms. Differences with *p* < 0.05 were considered statistically significant. To obtain additional information from the above functional annotations, the unigenes were also aligned with other functional databases, respectively.

## 3. Results

### 3.1. Genetic Makeup of the Biofilms

Statistical data of the metagenomic analysis obtained from three biofilms showed that the percentage of reads subject to trimming and quality filtering was consistent across libraries, and that the provided DNA was of good quality, as shown by the effective ratio high percentage of reads, validating the collection strategy (Table 2). Table 2 shows the original sequencing data (Raw data) by counting the number of concatenated sequences in each file, whereas the “Clean data” represents the sequences after preprocessing. The GC content was equivalent in the three biofilms, with SFNRad displaying the lowest percentage. Furthermore, SFNRad together with CRRad biofilms, showed a higher percentage of host DNA content. The removal of human genome using the software bowtie2 v2.2.0 showed an appropriate percentage of microbial DNA (Table 2). The data were validated by the higher number of microbial species detected (Table 3). Alpha Diversity Analysis supported the species’ diversity, richness, evenness, and sequencing depth within the collected biofilms (Table 3) (Supplementary Figure S1).

**Figure 1:**
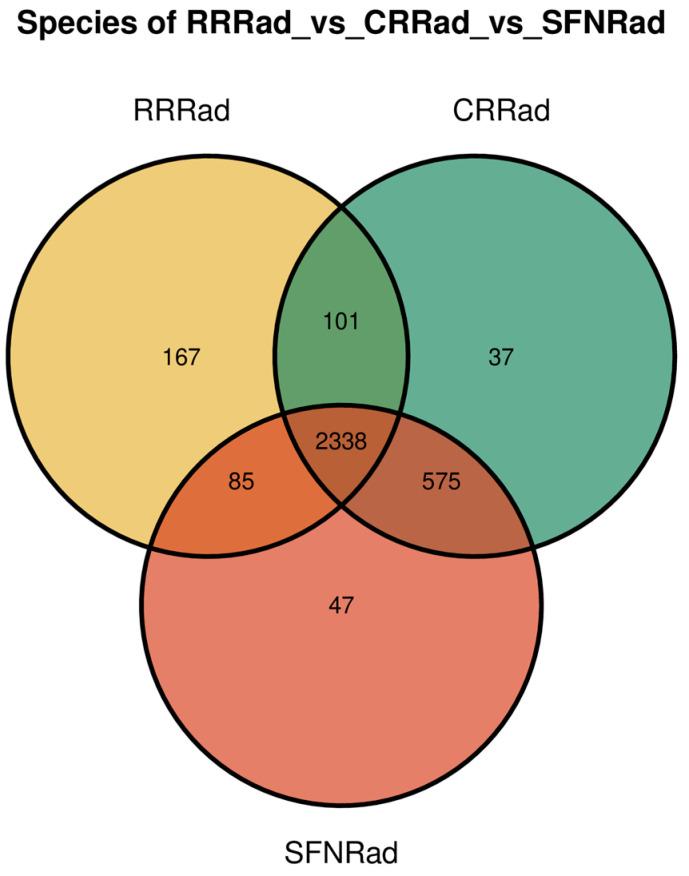
Venn diagram depicting the number of shared and unique microbial non-redundant unigenes at the species level among the three investigated biofilms.

The Shannon index derived from information entropy showed reliable diversity among samples. The program found 2616 species in RRRad, 3006 species in CRRad, and 3024 species in SFNRad biofilms (Table 3). The data show that the Simpson and Goods coverage were equally strong. For instance, the Simpson value was closer to 1.0 (maximum value) and the Goods coverage showed a high microbial coverage rate. Alpha diversity analysis using Chao1 also strongly found a similar number of species in the biofilms among individuals (Table 3). Figure 1 is a Venn diagram showing common and unique non- redundant unigenes at the species level among the three biofilms. The figure shows that the three biofilms shared 2338 unigene species. The RRRad biofilm possessed a higher number of unique species than those present on the other two biofilms (Figure 1).

### 3.2. Taxonomic Composition of the Biofilms

**Figure 2:**
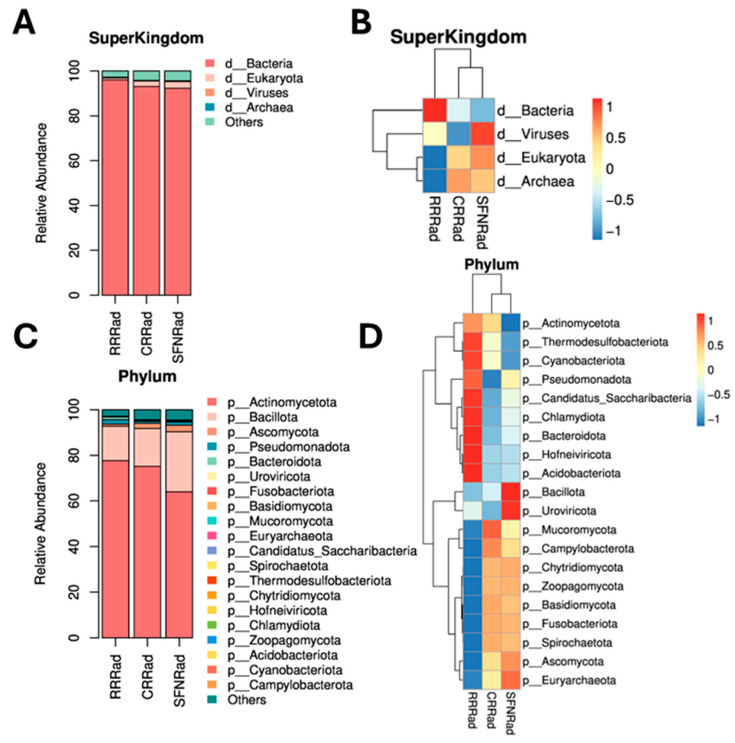
Panels (**A**,**C**) (StackedBar) and (**B**,**D**) (heatmap) show details of microbial communities’ diversity at the super kingdom (**A**,**B**) and phylum (**C**,**D**) levels on the investigated samples. The horizontal axis of panel (**A**,**C**) represents the sample names, and the vertical axis depicts the percentage of species abundance. The left sides of the panels (**B**,**D**) denote significant Spearman correlations (*p* < 0.05) between microbial communities and interrogated specimens. The top association in panels (**B**,**D**) indicates divergences or similarities in microbial communities between specimens. Panel (**B**,**D**) use color to reflect changes on the data information. In the latter panels, species and functional classification, or samples with similar abundance patterns (such as CRRad and SFNRad) are grouped together.

Using DIAMOND software, the unigene protein sequences were aligned with the NR_meta library. The best index for each unigene was selected as the species classification. Combined with the NCBI taxonomic system (which describes the correspondence between the sequence gi number and the taxonomic ID taxid), specific-species annotation information at different taxonomic levels were obtained (Supplementary Figure S1). The unigenes present in the three biofilms were assigned to four kingdoms; bacteria appeared more abundant, followed by eukaryotes, viruses, archaea, and others (Figure 2; Table 4). At the kingdom, phylum, family, and species levels, numerous microbial species were found (Figure 2). For instance, at the phylum level, the following microbial communities were more frequently detected: Actinomycetota, Bacillota, Ascomycota, Pseudomonadota, Bacteroidota, Uroviricota, Fusobacteriota, and many others, with the Actinomycetota being the most abundant (Figure 2C). The percentage of microbes at the phylum level detected in the biofilms investigated is shown in Table 4. Interestingly, the presence of a high number of unclassified taxa was found (Table 5). Of interest, also, was the finding of several viruses, Chytridiomycota, archaeal, and unclassified species at very low percentages (Table 5).

**Figure 3:**
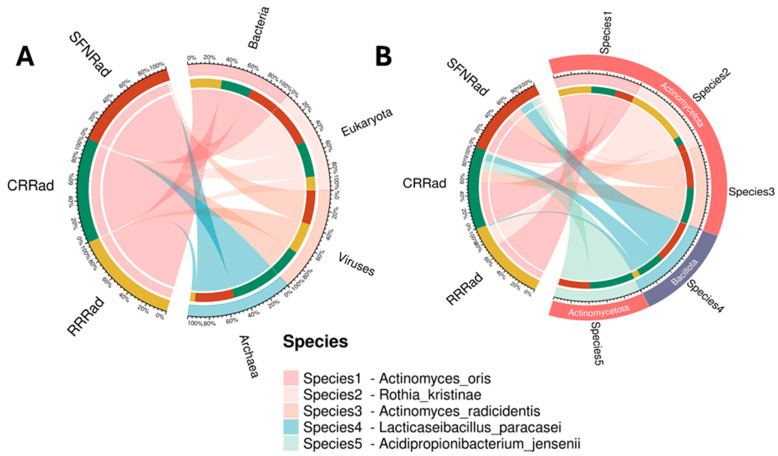
Panels (**A**,**B**) show two Circos plots depicting correspondence between sample microbe domains, kingdom (**A**), and species (**B**). The wider the width of the color biofilms on the right (corresponding to each biofilm on the left), the higher the abundance, and vice versa. The lower section of the figure shows the species names depicted by color in panel (**B**) right section.

The Circos plots showed a visualization at the kingdom level of the most abundant microbial communities in the three biofilms (Figure 3A). Figure 3B is a Circos plot showing the most abundant bacteria species in the Actinomycetota. The higher percentage of species included *Acidipropionibacterium jensenii* (RRRad = 0%, CRRad = 12%, and SFNRad = 8%).

*Actinomyces oris* (RRRad = 70%, CRRad = 62%, and SFNRad = 60%), *Actinomyces radicidentis* (RRRad = 0.5%, CRRad = 22%, and SFNRad = 27%), and *Rothia kristianae* (RRRad = 28.5%, CRRad = 2%, and SFNRad = 3%) (Figure 3B). Within the second most abundant bacterial phylum, the Bacillota (Figure 2C), *Lacticaseibacillus paracasei* was found in high percentages (RRRad = 0.5%, CRRad = 2.5%, and SFNRad = 2%) (Figure 3B). Less abundant unigenes belonged to unusual prokaryotes, eukaryotes, viruses, and unidentified species. From a long list of unusual species, the most prominent were *Mycobacterium tuberculosis*, *Mycobacterium* sp., *Candida* (*Candidozyma*) *auris*, as well as other uncommon prokaryotes, eukaryotes, and viruses in oral devices (Table 6). The complete list of species (3350) uncovered in the three biofilms can be found in Supplementary Figure S1.

### 3.3. Functional Analysis of Metabolic Pathways

**Figure 4:**
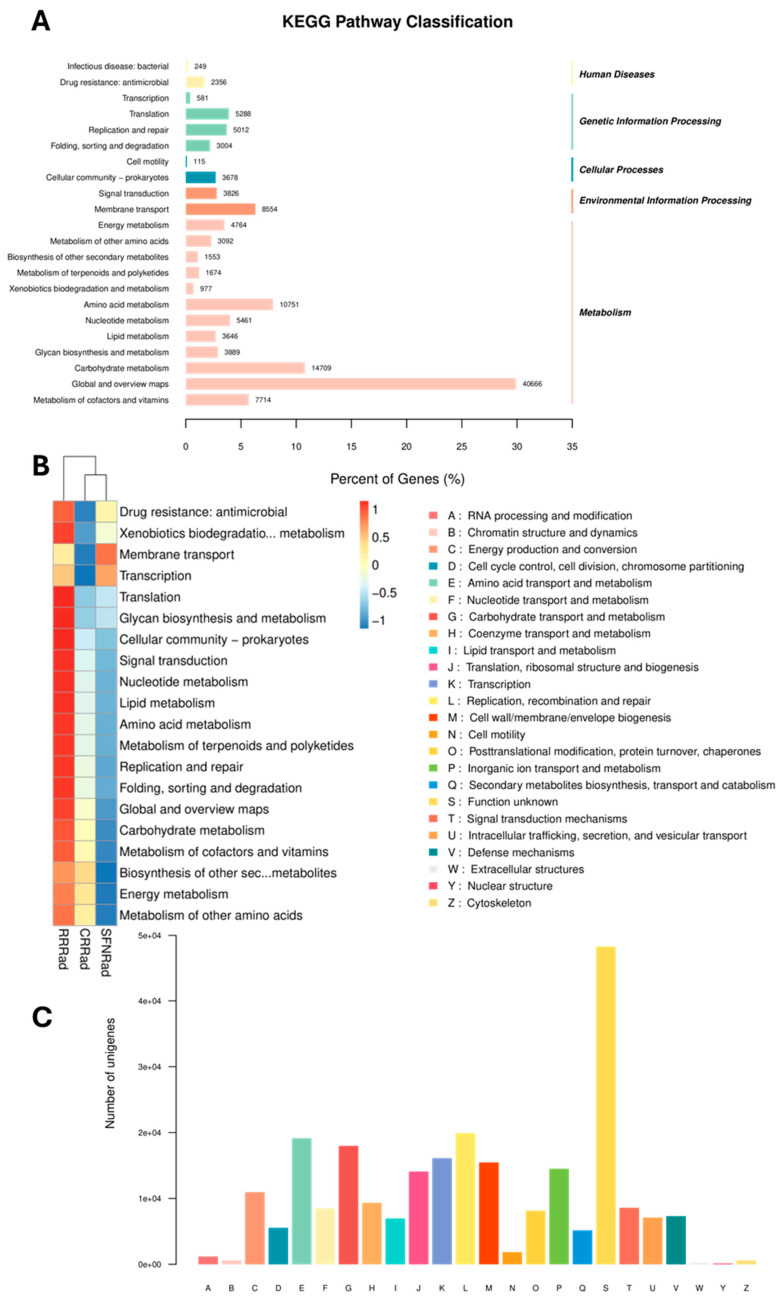
Functional unigene prediction of the microbial communities in the three biofilms. Panel (**A**) shows classification based on Kyoto Encyclopedia of genes (KEGG) database showing differential abundance levels of the annotated orthologous unigenes. Panel (**B**) depicts a heatmap vertical comparison of the three sampled species functional classification. Samples with similar abundance patterns are grouped together. Panel (**C**) shows an eggNOG classification chart. On the horizontal axis, each COGFunctionalCategory is depicted (A to Z); the vertical axis contains the number orthologous unigenes annotated (0 to 5 × 10^4^).

Using the KEGG database, all nonredundant unigenes were grouped according to their functional pathways (Figure 4A,B). The most annotated functional unigenes were those related to: (1) global and overview maps such as carbohydrate metabolism, amino acid metabolism, membrane transport, and the metabolism of cofactors, vitamins and others; (2) environmental information processing like signal transduction and membrane transport; (3) genetic information processing, including translation, replication and repair folding, sorting and degradation, and others; (4) cellular processes such as cell motility; and (5) human diseases, drug resistance, and virulence factors. According to the eggNOG annotations, the most abundant functional unigenes were those with unknown functions (S, 5.0 × 10^4^), followed by replication, recombination, and repair (L, 2.0 × 10^4^), amino acid transport and metabolism (E, 2.0 × 10^4^), carbohydrate transport and metabolism (G, 2.0 × 10^4^), and others (Figure 4C).

### 3.4. Carbohydrate Enzymes Profiles

**Figure 5:**
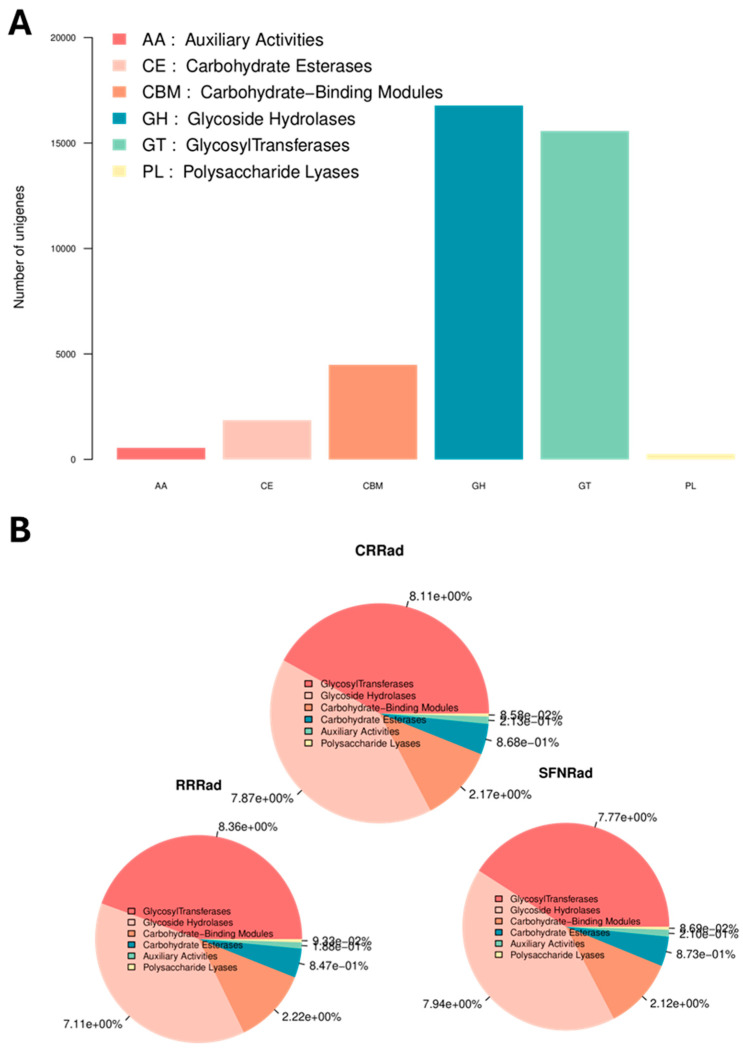
Panel (**A**) shows the number of the non-redundant unigenes assigned to CAZy modules with high unigenes numbers for glycosidase hydrolases and glycosyl transferases. Panel (**B**) depicts the abundance of CAZy modules among the three biofilms.

Among the non-redundant unigenes found on the three biofilms, the majority were assigned by the CAZy database to several groups of enzymes (Figure 5A,B). Among the most abundant were unigenes for enzymes with glycoside hydrolases (GH) and glycosyl transferase (GT) activities, followed by carbohydrate-binding modules (CBM), carbohydrate esterases (CE) and others (AA and PL) (Figure 5A). The abundance of the unigenes encoding carbohydrate enzymes is shown in Figure 5B.

### 3.5. Antibiotic Resistance Genes (ARGs)

**Figure 6:**
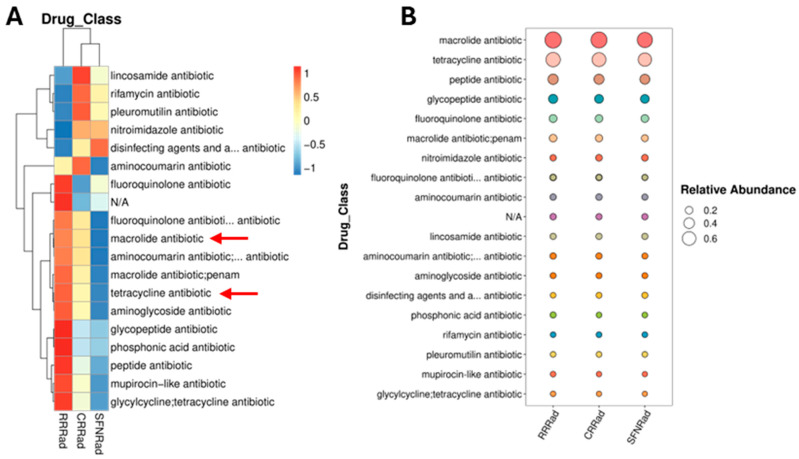
Panel (**A**) shows a heatmap chart from the CARD database depicting antibiotic resistance unigenes found in the three biofilms. The left of panel (**A**) denotes the Spearman correlation between antibiotic functional genes in the interrogated specimens; the top association indicates divergences or similarities of the genes in the investigated biofilm. Panel (**B**) is a bubble plot showing resistance unigenes (**left**) and their relative abundance (**right**). In panel (**B**), macrolide and tetracycline ARGs were the most abundant of the three biofilms, depicted with reds arrows in panel (**A**).

Using the Comprehensive Antibiotic Resistance Database (CARD) (https://card.mcmaster.ca/home, accessed 29 May 2025), several antibiotic-resistant genes and related gene-coding peptides and proteins were found in the three biofilms (Figure 6A,B). Macrolide and tetracycline ARGS were the most abundant orthologous genes detected in the biofilms (Figure 6A red arrows and Figure 6B), followed by less abundant ARGs (Figure 6B). The biofilm with the most antibiotic-resistant genes was RRRad, including the highest number of ARGs resistant to macrolide and tetracycline antibiotics (Figure 6A red arrows). In contrast, CRRad and SFNRad biofilms, in general, showed few ARGs. The finding that the latter two biofilms possess the compounds lacosamide, rifamycin, and pleuromutilin, is also relevant to this study.

Nitroimidazole, disinfecting agents, antiseptics, and fluoroquinolone resistance unigenes absent in RRRad biofilm (Figure 6A). The ARGs found in the three biofilms showed several resistance mechanisms, some of which were related to antibiotic efflux pump, and were the most abundant group of ARGs (Figure 6). One of the resistance mechanisms showed 100% identity with tetracycline antibiotic efflux mechanism, which is part of the gene family related to the major facilitator of antibiotic efflux pump. The latter resistance mechanism was present in the RRRad biofilm but absent in the other two biofilms. 

## 4. Discussion

To our knowledge, this is the first metagenomic study investigating the microbial communities present in obturator biofilms. The low number of specimens used in this study reflects the small population of individuals bearing these devices, and more importantly, the reluctance of many patients to share a personal device for the emotional and physical discomfort that such an action would entail. Several studies on patients with obturator prostheses showed their unwillingness to cooperate when the methodology required the device’s removal, even briefly. These studies showed that after the removal of the devices, basic functions, such as palatal seal, swallowing of saliva, and speech intelligibility, could cease, increasing functional and aesthetic discomfort in an already vulnerable population [17,18] As discussed by Rumsey and Harcourt [19] and Cunningham [20], facial differences and public exposure of the condition exacerbate social anxiety, restricting the availability and participation of these patients in studies that require the removal of their prostheses. In this context, the temporary removal of the prosthesis, even for minutes, to collect the biofilm, constitutes an ethical and operational barrier to recruitment, since participation cannot be compelled. Therefore, refusal is a common outcome [17]. It is likely that other investigators working with this population of patients will encounter similar difficulties, stemming from the same functional limitations and psychosocial constraints [19,20]. Consequently, the findings of this study, even in a small population, present a unique opportunity to understand the composition and function of the microbial communities developed on obturator biofilms.

Despite the encountered constraints, we identified >3000 species, a number that exceeds by several fold the typical number obtained by metagenomic surveys on dentures, dental plaques, or implants [15,21,22,23,24,25,26]. In addition to the high taxonomic diversity encountered in this study, several metabolic pathways and >100 ARGs, mainly macrolide and tetracycline efflux pumps and several genes encoding putative virulence factors, were found. Traditional microbiological analysis of unsuccessful implants and dentures identified 12–77 species [20,21,22,23,24,25,26,27,28,29,30], whereas studies of healthy dental plaque metagenomics found 288 species in 113 samples [22,25,26]. In contrast, the microbial biofilm communities found in this study showed an average of between 2616 and 3024 different species. Interestingly, earlier studies reported a predominance of *Actinomyces*, *Rothia* and *Acidipropionibacterium*. These genera also were found in high percentages in our study, suggesting their strong affinity to hard surfaces, as described in previous denture, enamel, and dentin studies [15,21,22,25,26]. The contrasting differences in the number of species encountered in our study versus those in the oral literature, including denture devices, resides in the biofilm collection strategies and the fact that our analysis included not only a high percentage of unigenes, but species detected at ≤1.0% and lower. We strongly believe that this strategy will uncover unusual pathogens with slow division cycles yet to be reported in oral biofilms. For instance, during large-scale studies, low numbers of common oral microbiota were encountered [11,12,13,14,15,22,31]. Thus, by focusing on a large number of individuals, those studies likely missed oral microbes that are present at very small percentages. Incidentally, a recent study found that the composition of oral biofilms formed on dental implants are complex, diverse, and dynamic [32]. Although Dieckow et al. [32] found 371 species at ≥1.0%, their data showed 100 unclassified new microbes, and several types of viruses, a finding that is in agreement with our study. The presence of more than 2600 species on the three biofilms, including the presence of highly pathogenic and opportunistic species, suggest that these devices hold rich, diverse, and unique microbial communities warranting further investigation (Table 5).

Despite previous findings of *M. tuberculosis* on body prostheses [33,34,35,36,37,38,39], in our study, this highly pathogenic species was detected at a very low percentage. Although shotgun metagenomics could target multiple DNA signals [11,12,13,14,15], viability, active colonization, or infection are not well supported. The detection of this pathogen at low percentages may also reflect transient deposition, misclassification among closely related taxa, or contamination introduced during sampling or processing A similar situation can be found with *Candida auris*, an emerging multidrug-resistant opportunistic fungal pathogen [40,41,42,43]. In this study, unigenes classified as *C. auris* were detected also at very low percentages across the investigated biofilms. Therefore, these findings should be interpreted cautiously and confirmed using targeted approaches (e.g., species-specific qPCR from independent extractions).

The differences and similarities in species percentages in each of the investigated prostheses are probably related to their anatomical locations within the mouth, and their connection with one or more nearby open cavities, developed during invasive surgical procedures. The data in this study showed that the direct communication of the oral cavities with adjacent anatomical structures, such as the maxillary sinus, nasal, and orbital cavities, resulting from previous surgeries, could modify the local microenvironment and, consequently, impact the composition and structure of the microbial communities on the biofilms investigated (Figure 1) [17,18]. Moreover, the numerous species found in this study, absent from previous oral studies using similar methodologies [11,12,13,14,15,22,29,30,31,44,45,46], imply that the current microbial composition on these devices may be only the tip of the iceberg [29,30,44,45,46]. Conversely, our data show that the presence of ARG genes may have strong implications on the oral health of patients receiving antibiotic management, especially with macrolides and tetracyclines.

## 5. Limitations of the Study

Although the study provides significant contributions to the understanding of microbial communities in the biofilms developed on obturator prostheses, it also has several limitations. For instance, the sample size is relatively small, impacting on the generalizability of the results. The analytical cross-sectional nature of the study limits the ability to capture biofilm changes over time. Therefore, these findings are preliminary. Further analyses are necessary to validate the role of these microbial communities on obturator prosthesis biofilms and explore their prevalence in health and disease, ensuring proper public health strategies in patients wearing obturator prostheses. Moreover, the discomfort felt by patients when providing their devices during this study constitutes an ethical and operational barrier to recruitment, an issue that has a negative impact on the number of samples obtained for this study. In addition, key clinical and prosthesis-related variables (exact timing of radiotherapy relative to sampling, prosthesis fabrication/delivery dates, and detailed design features such as hollow- versus solid-bulb configuration) were not available, limiting clinical stratification.

## 6. Conclusions

This preliminary shotgun metagenomics study suggests that maxillary obturator prostheses may support biofilms with highly diverse, multi-kingdom microbial communities and a broad repertoire of antimicrobial-resistance gene signatures. Our data indicates that obturator-associated biofilms could act as reservoirs of different microbial communities with potential clinical relevance. Larger longitudinal studies integrating detailed clinical/prosthetic variables and targeted validation of low-abundance taxa are needed to clarify associations with mucosal health in patients with obturator protheses.

## Figures and Tables

**Table 1:** Participants’ clinical and prosthesis-related features.

Patients	Sex	Age (Years)	Dentition	Therapy	Indication/ Diagnosis	Surgery/ Defect	Prosthesis
RRRad	Female	72	Partially dentate	CT: No RT: No	Carcinoma	Limited maxillectomy Central hard palate	Removable partial obturator (acrylic resin + metal framework) ≥1 year use
CRRad	Male	79	Partially dentate	CT: No RT: Yes (2022)	Ameloblastoma	Subtotal maxillectomy Right infrastructure	Removable partial obturator (acrylic resin + metal framework) ≥1 year use
SFNRad	Male	42	Partially dentate	CT: No RT: No	Hospital-acquired mucormycosis	Subtotal maxillectomy Lateral infrastructure	Removable partial obturator (acrylic resin + metal framework) ≥1 year use

CT = Chemotherapy; RT = Radiotherapy.

**Table 2:** Sequencing data quality control.

Sample	Raw Data		Clean Data		Valid Data		Effective Ratio %	GC %	Host Genome
	Reads	Base	Reads	Base	Reads	Base			
RRRad	38,180,952	5.73 G	37,289,430	5.59 G	35,867,396	5.38 G	97.67	61.83	3.79%
CRRad	40,762,654	6.11 G	39,056,280	5.86 G	25,515,768	3.83 G	98.42	55.76	34.51%
SFNRad	50,624,642	7.59 G	49,192,604	7.38 G	18,851,340	2.83 G	97.17	48.37	61.58%

**Table 3:** Statistical alpha diversity indices.

Sample	Observed Species	Shannon Index	Simpson Index	Chao1	Goods Coverage
RRRad	2616	4.23	0.86	2673.66	1.0
CRRad	3006	5.09	0.92	3076.05	1.0
SFNRad	3024	5.48	0.94	3084.79	1.0

**Table 4:** Percentages of unigene abundance among the three biofilms at the kingdom level.

Super Kingdom	RRRad	CRRad	SFNRad
Bacteria	95.99	93.09	92.32
Others	2.76	4.18	4.37
Eukaryota	0.93	2.43	2.94
Viruses	0.32	0.29	0.35
Archaea	1 × 10^−7^	0.02	0.01

**Table 5:** Percentages of microbial diversity unigenes by phylum using alpha diversity analysis. Unclassified species are highlighted in red. Low percentages are expressed in relative abundance.

Phylum	RRRad	CRRad	SFNRad
Actinomycetota	77.65	75.17	63.95
Bacillota	15.09	16.68	25.79
Unclassified	2.76	4.18	4.76
Ascomycota	0.92	2.30	2.87
Pseudomonadota	2.01	0.83	1.42
Bacteroidota	1.19	0.26	0.35
Uroviricota	0.27	0.25	0.32
Fusobacteriota	0.01	0.12	0.21
Virus unclassified	0.04	0.03	0.03
Basidiomycota	0.01	0.03	0.03
Eukaryote unclassified	296 × 10^−7^	0.04	0.03
Mucormycota	267 × 10^−7^	0.01	0.05
Saccharibacteria	0.01	0.01	0.01
Spirochaetota	173 × 10^−7^	0.01	0.01
Chytridiomycota	27 × 10^−7^	0.01	0.01
Hofneiviricota	0.01	193 × 10^−7^	22 × 10^−7^
Acidobacteriota	0.01	44 × 10^−7^	122 × 10^−7^
Archaea unclassified	77 × 10^−7^	35 × 10^−7^	5 × 10^−7^

**Table 6:** Relative abundance of unigenes in uncommon microbes found in the investigated biofilms.

Species Names	RRRad	CRRad	SFNRad
*Mycobacterium tuberculosis*	3 × 10^−4^	3 × 10^−4^	4 × 10^−4^
*Mycobacterium* sp. AT1	0	264 × 10^−7^	261 × 10^−7^
*Candida* (*Candidozyma*) *auris*	29 × 10^−7^	168 × 10^−7^	212 × 10^−7^
*Nocardia nova*	17 × 10^−7^	14 × 10^−6^	153 × 10^−7^
*Mycobacterium* phage OkiRoe	0	91 × 10^−7^	103 × 10^−7^
*Rhizophagus clarus (mycorrhizae)*	8 × 10^−7^	104 × 10^−7^	73 × 10^−7^

## Data Availability

The metagenome data collected during this study have been deposited at the National Center for Biotechnology Information (NCBI) under the accession number: PRJNA1346426.

## Supplementary Materials

The following supporting information can be downloaded at: https://www.mdpi.com/article/10.3390/pathogens15020221/s1, Figure S1: Panel A shows the length distribution of non-redundant Unigenes. Panel B is an Alfa Diversity rarefaction curve showing the number of species richness on the three investigated biofilms.
