# Supplementary material for: Microbial Diversity and Composition Uncovered on Obturator Prosthesis Biofilms: Exploratory Findings from a Pilot Study
Source: Pathogens. 2026 Feb 16;15(2):221. doi: 10.3390/pathogens15020221 (PMC12943561; doi:10.3390/pathogens15020221)
Supplement: Supplementary file 1 [file pathogens-15-00221-s001.zip › pathogens-4039456-supplementary.pptx]

## Slide 1
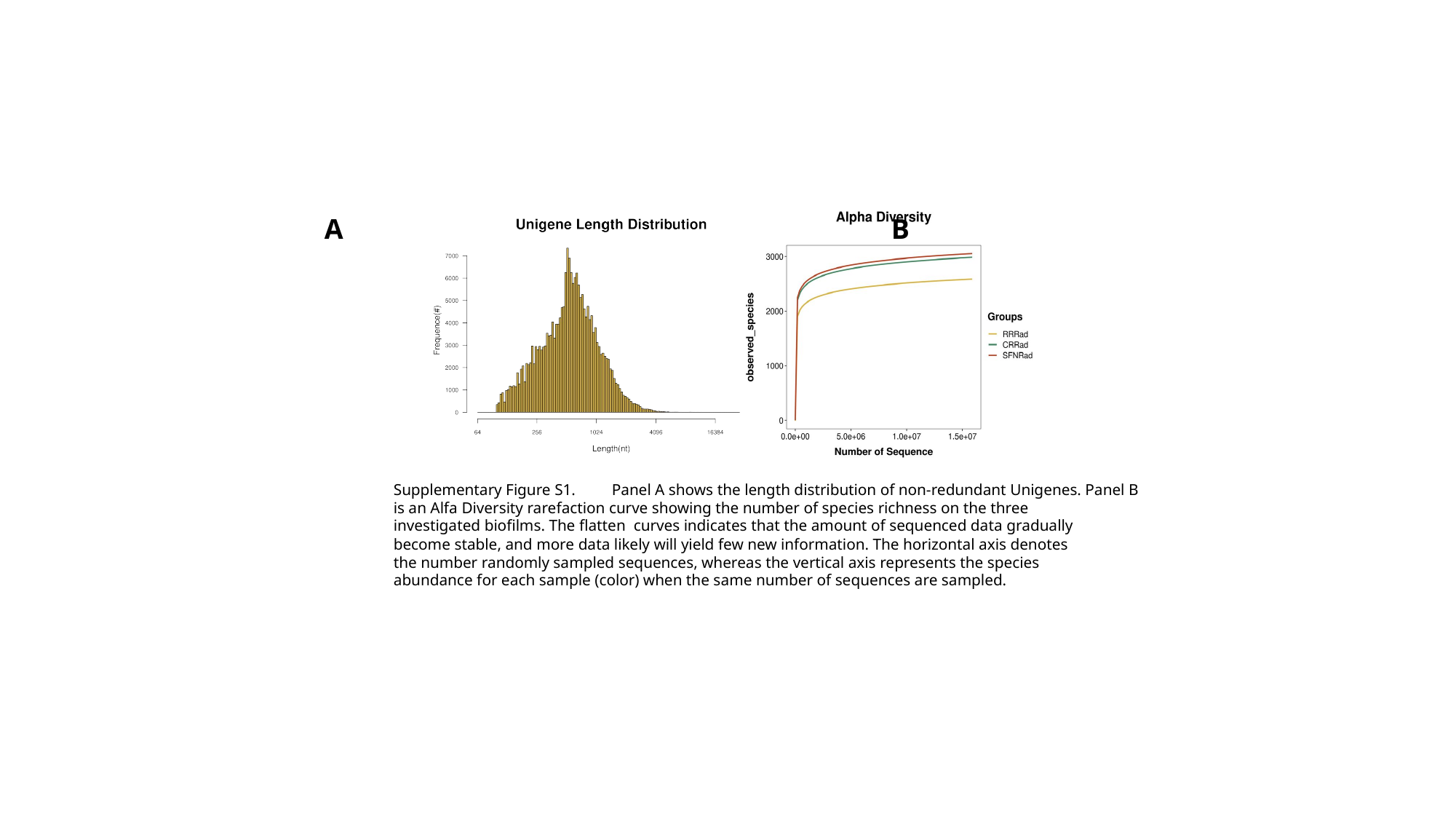

A					 B
Supplementary Figure S1.	Panel A shows the length distribution of non-redundant Unigenes. Panel B
is an Alfa Diversity rarefaction curve showing the number of species richness on the three
investigated biofilms. The flatten curves indicates that the amount of sequenced data gradually
become stable, and more data likely will yield few new information. The horizontal axis denotes
the number randomly sampled sequences, whereas the vertical axis represents the species
abundance for each sample (color) when the same number of sequences are sampled.
